# Mechanotransduction in neutrophil: Mechanosensing and immune function regulation

**DOI:** 10.1016/j.mbm.2025.100157

**Published:** 2025-09-07

**Authors:** Wenying Zhao, Jin Wang, Jing Wang

**Affiliations:** Shanghai Institute of Immunology, Shanghai Jiao Tong University School of Medicine, Shanghai, China

**Keywords:** Mechanotransduction, Neutrophil, Mechanosensing, PIEZO1, TRPV4

## Abstract

Immune cells sense and transduce mechanical signals such as stiffness, stretch, compression, and shear stress. In the past few years, our understanding of the mechanosensitive signaling pathways in myeloid cells has significantly expanded, especially in monocytes, macrophages, and dendritic cells. Recently, the mechanobiological regulation of neutrophil function has been deciphered. Mechanical signals from tissue-derived shear stress and cellular deformation tension reprogram neutrophil transcription via GEF-H1, PIEZO1, and TRPV4 pathways, modulating neutrophil functions in homeostasis and trans-endothelial migration. Understanding these force-dependent processes provides novel insights into neutrophil plasticity and highlights potential therapeutic strategies and approaches for inflammatory and infectious diseases.

## Graphical abstract

**Figure:**
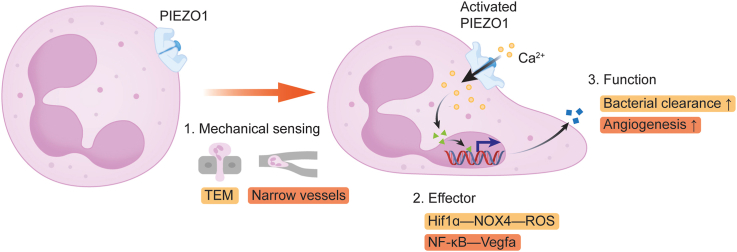

Mechanobiology is transforming our understanding of immune regulation by revealing how physical forces influence immune cell functions in health and disease. In the past 20 years, research has shown that a cell's physical environment and the forces exerted by cells and tissues are crucial in both healthy and diseased states. In response, cells adapt by converting physical signals into biochemical signals and changes in gene expression through mechanotransduction pathways. Neutrophils are the pivotal innate immune cells in early immune responses. As circulating cells, neutrophils encounter and respond to varying physical microenvironments, adapting their behavior to navigate different tissues during immune responses. A recent study provides compelling evidence that trans-endothelial neutrophil activates bactericidal function via mechanosensing.^1^ This study highlights the role of mechanosensitive PIEZO1 sensing elevated mechanical tension and triggering intracellular Ca^2+^ influx upon transmigrating through the restrictive endothelial adherens junctions (AJs). Further investigation revealed that PIEZO1 signals enhance neutrophil bactericidal activity by transcriptional upregulation of *Nox4* via Hif1a stabilization. These findings underscore the key role of mechanical regulation of immune responses and suggest the possibility of therapy in bacterial-induced tissue injury.

Neutrophils also possess the ability to sense mechanical forces within the tissue microenvironment under physiological conditions. A recent study from our group demonstrated that deformation of neutrophils navigating through narrow pulmonary vessels activates the mechanosensitive ion channel PIEZO1 and the downstream ERK/NF-κB pathway, driving their transcriptional reprogramming to acquire proangiogenic functions, thereby maintaining pulmonary vascular homeostasis and enhancing anti-infection capabilities.^2^ Recent studies have highlighted the remarkable plasticity and heterogeneity of neutrophils. This indicated that neutrophils exhibit diverse phenotypes and functions, adapting to microenvironmental cues through complex signaling pathways, including mechanotransduction.

These two studies together reveal that neutrophil deformation within confined microenvironments plays a critical role in regulating neutrophil phenotypes and key functions by converting membrane tension forces into biochemical signals (Fig. 1).

**Fig. 1:**
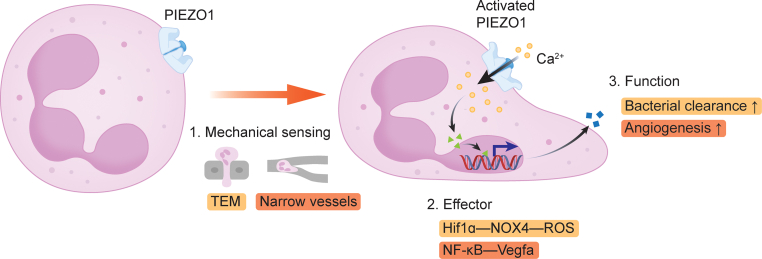
Schematic of neutrophil mechanical sensing and function regulation via PIEZO1. Neutrophils sense mechanical forces to regulate antibacterial responses and pro-angiogenic functions. PIEZO1 serves as a critical mechanosensor in neutrophil mechanotransduction.

A limited and conserved set of sensors and pathways in myeloid cells governs their responses to diverse environmental cues.^3^ One of the best characterized mechanotransducers is mechanically gated cation channel PIEZO1 and the transcription factors YAP/TAZ.^4^ Cyclical hydrostatic pressure engages PIEZO1 in innate immune cells and initiates an inflammatory transcriptional program through endothelin-1 (EDN1) and stabilization of HIF-1α.^5^ PIEZO1 deficiency in innate cells markedly reduced pulmonary inflammation in infection and fibrotic models. In macrophages, matrix stiffening upregulates PIEZO1 expression and induces PIEZO1-dependent Ca^2+^ influx, which promotes nuclear YAP localization and accumulation to drive an M1 transcriptional program with increased TNF-α and IL-6 and decreased IL-10.^6^ Pharmacologic inhibition of YAP reversed the stiffness-induced M1 polarization and improved implant integration.^6^ TRPV4 is another Ca^2+^-permeable TRP channel activated by osmotic, stretch, and matrix stiffness cues. Increased substrate stiffness (*in vitro* and in fibrotic skin) drives M1 polarization in a TRPV4-dependent manner: stiff matrices increased M1 marker expression, including TNF-α, IL-1β, IL-6, IL-12, and iNOS.^7^ TRPV4 activation, cooperating with LPS-induced signals enhances macrophage bacteria phagocytosis and oxidized-LDL uptake resulting in foam-cell formation.^8^^,^^9^ In addition, adhesion receptors mediate sustained mechanical sensing through focal adhesion kinase (FAK)/Src, Rho GTPases and actomyosin tension, which feed the Hippo pathway effectors YAP and TAZ. Adhesive microenvironment and substrate stiffness control macrophage inflammatory output (LPS-stimulated secretion of TNF-α, IL-6, and IL-10) through YAP/TAZ.^10^ In dendritic cells, substrate stiffness and integrin engagement regulate surface maturation markers (e.g., CD83), podosome assembly, chemokine receptor CCR7 expression, and migratory competence that directly affect antigen-presentation and T-cell priming.^11^ Important open questions remain regarding pathway crosstalk, cell-type specificity, and *in vivo* spatiotemporal dynamics under complex tissue mechanics. Elucidating these interactions will be critical to translate mechanobiology into therapies for infection, fibrosis, and biomaterial design.

Nested within this broader context, neutrophils have emerged as uniquely adaptable to mechanical forces, reflecting their role as first responders in fluid and tissue environments. Neutrophils, the most abundant type of leukocyte in blood and pivotal players in early immune responses, have long been understood to migrate and activate under the influence of chemoattractants, such as fMLP, IL-8, and pathogen-associated molecular patterns. However, emerging evidence now reveals that mechanical forces also serve as a significant regulator of neutrophil function.

Early recognition of mechanical regulation in neutrophils centered on selectin–ligand behavior, because neutrophils and other leukocytes crawl and transmigrate optimally in the presence of shear stress under conditions of blood flow. Fine et al. identified the microtubule-associated guanine exchange factor GEF-H1 as a shear-specific mechanosensor.^12^ Under shear, GEF-H1 relocates to the uropod, activating RhoA and ROCK to induce actomyosin contraction, enabling crawling and trans-endothelial migration (TEM). Importantly, GEF-H1 is dispensable for chemokine-driven chemotaxis, highlighting the mechanochemical specificity of this pathway. And selectin catch-bonds under higher shear flow mechanically transduce signals to activate integrins (LFA-1/Mac-1), enabling neutrophil arrest on inflamed endothelium to counteract increasing shear forces.^13^

Recently, PIEZO1 was brought into the spotlight for neutrophil mechanotransduction via shear stress in blood flow. A study demonstrated that exposure to high shear (∼80 ​dyne/cm^2^) activates PIEZO1 in human circulating neutrophils, eliciting rapid calcium influx, calpain activation, and cytoskeletal remodeling, ultimately triggering histone H3 citrullination and robust NETosis, even in the absence of chemical NETs inducers like PMA or LPS.^14^ Remarkably, NETs formation scaled directly with shear stress magnitude, and PIEZO1 pharmacologic inhibition or genetic knock-down markedly attenuated this mechanosensitive NETs release. Further extending the role of Piezo1 in neutrophil function, a study exploring atherosclerosis in ApoE^−/−^ mice found that low shear stress (LSS) in carotid arteries paradoxically downregulated PIEZO1 expression, causing reduced Ca^2+^ influx, increased HDAC2 activity, enhanced ROS, and increased NETosis, leading to endothelial apoptosis and aggravated plaque progression.^15^ Pharmacologically restoring PIEZO1 with agonist Yoda1 could reverse NETosis and attenuate plaque formation, spotlighting a PIEZO1–HDAC2 axis of mechanical regulation in chronic vascular inflammation.

PIEZO1 activation during neutrophil deformation regulates effector functions. During in vivo migration, neutrophils inevitably traverse constrictions narrower than their diameter (∼10μm). For instance, sustained mechanical compression from pulmonary capillaries (∼5μm diameter) triggers PIEZO1-mediated Ca^2+^ influx, leading to unique transcriptional profiles including high-expressed proangiogenic and inflammatory mediators (VEGFA, IL1B, CXCL1/2, and IL6).^2^ As neutrophils squeeze through AJs in inflammation, transient PIEZO1 opening increases calcineurin activity and stabilizes HIF-1α, leading to *Nox4* upregulation and amplification of the NADPH oxidase-mediated respiratory burst.^1^ In mouse models of *Pseudomonas aeruginosa* lung infection, conditional PIEZO1 deletion in neutrophils elevated bacterial burden.

Although these findings solidify PIEZO1's central role in neutrophil mechanobiology, TRPV4 also appears to contribute. In mice undergoing myocardial ischemia–reperfusion injury, TRPV4 expressed on neutrophils was shown to mediate Ca^2+^ influx in response to mechanical and chemotactic stimuli, promoting reactive oxygen species (ROS) generation, myeloperoxidase release, and migration toward fMLP.^16^ TRPV4 antagonists reduced neutrophil-mediated cardiac injury, suggesting a role for TRPV4 in mechanical and inflammatory sensing by neutrophils. It might be a novel therapeutic target for myocardial ischemia-reperfusion injury and other neutrophil-mediated inflammatory diseases.

Integrating these findings reveals two key mechanotransduction modules that coordinately regulate neutrophil behavior and functions during tissue homeostasis and TEM: (i) a shear-driven module comprising GEF-H1/RhoA/ROCK pathway and selectin–integrin catch-bonds; (ii) a mechanosensitive ion channel module involving PIEZO1 and TRPV4-mediated calcium signaling. Despite these advances, major knowledge gaps persist. While most studies have focused on vascular or pulmonary settings, numerous other physiological and pathological settings remain unexplored. The intricate integration of mechanical and chemical cues and how PIEZO1/Ca^2+^ signaling influences transcription are still unclear. Additionally, most in vitro microfluidic devices fail to fully replicate the multiscale mechanical complexity of physiological microenvironments, highlighting the need for novel techniques to quantify and manipulate mechanical forces under in vivo conditions. Neutrophil mechanobiology offers critical insights into bacterial clearance, thrombosis prevention, and other neutrophil-associated pathologies. Further investigation of mechanosensing mechanisms in neutrophils and other immune cells holds substantial clinical potential.

## CRediT authorship contribution statement

**Wenying Zhao:** Writing – original draft. **Jin Wang:** Writing – review & editing. **Jing Wang:** Funding acquisition, Supervision, Writing – review & editing.

## Ethical approval

This study does not contain any studies with human or animal subjects performed by any of the authors.

## Declaration of competing interest

The authors declare that they have no known competing financial interests or personal relationships that could have appeared to influence the work reported in this paper.
